# Distinct Effects of Escitalopram and Vortioxetine on Astroglial L-Glutamate Release Associated with Connexin43

**DOI:** 10.3390/ijms221810013

**Published:** 2021-09-16

**Authors:** Takashi Shiroyama, Kouji Fukuyama, Motohiro Okada

**Affiliations:** Department of Neuropsychiatry, Division of Neuroscience, Graduate School of Medicine, Mie University, Tsu 514-8507, Japan; takashi@clin.medic.mie-u.ac.jp (T.S.); k-fukuyama@clin.medic.mie-u.ac.jp (K.F.)

**Keywords:** 5-HT receptor, 5-HT transporter, astrocyte, connexin43, depression

## Abstract

It has been established that enhancement of serotonergic transmission contributes to improvement of major depression; however, several post-mortem studies and experimental depression rodent models suggest that functional abnormalities of astrocytes play important roles in the pathomechanisms/pathophysiology of mood disorders. Direct effects of serotonin (5-HT) transporter inhibiting antidepressants on astroglial transmission systems has never been assessed in this context. Therefore, to explore the effects of antidepressants on transmission associated with astrocytes, the present study determined the effects of the selective 5-HT transporter inhibitor, escitalopram, and the 5-HT partial agonist reuptake inhibitor, vortioxetine, on astroglial L-glutamate release through activated hemichannels, and the expression of connexin43 (Cx43), type 1A (5-HT1AR) and type 7 (5-HT7R) 5-HT receptor subtypes, and extracellular signal-regulated kinase (ERK) in astrocytes using primary cultured rat cortical astrocytes in a 5-HT-free environment. Both escitalopram and 5-HT1AR antagonist (WAY100635) did not affect basal astroglial L-glutamate release or L-glutamate release through activated hemichannels. Subchronic (for seven days) administrations of vortioxetine and the 5-HT7R inverse agonist (SB269970) suppressed both basal L-glutamate release and L-glutamate release through activated hemichannels, whereas 5-HT1AR agonist (BP554) inhibited L-glutamate release through activated hemichannels, but did not affect basal L-glutamate release. In particular, WAY100635 did not affect the inhibitory effects of vortioxetine on L-glutamate release. Subchronic administration of vortioxetine, BP554 and SB269970 downregulated 5-HT1AR, 5-HT7R and phosphorylated ERK in the plasma membrane fraction, but escitalopram and WAY100635 did not affect them. Subchronic administration of SB269970 decreased Cx43 expression in the plasma membrane but did not affect the cytosol; however, subchronic administration of BP554 increased Cx43 expression in the cytosol but did not affect the plasma membrane. Subchronic vortioxetine administration increased Cx43 expression in the cytosol and decreased it in the plasma membrane. WAY100635 prevented an increased Cx43 expression in the cytosol induced by vortioxetine without affecting the reduced Cx43 expression in the plasma membrane. These results suggest that 5-HT1AR downregulation probably increases Cx43 synthesis, but 5-HT7R downregulation suppresses Cx43 trafficking to the plasma membrane. These results also suggest that the subchronic administration of therapeutic-relevant concentrations of vortioxetine inhibits both astroglial L-glutamate and Cx43 expression in the plasma membrane via 5-HT7R downregulation but enhances Cx43 synthesis in the cytosol via 5-HT1AR downregulation. This combination of the downregulation of 5-HT1AR, 5-HT7R and Cx43 in the astroglial plasma membrane induced by subchronic vortioxetine administration suggest that astrocytes is possibly involved in the pathophysiology of depression.

## 1. Introduction

Several post-mortem studies reported a reduction in astrocyte density and an enlargement in size, as well as gap junctions/hemichannels in various cortical and subcortical regions, such as the frontal cortex, mediodorsal thalamus, caudate nucleus and locus coeruleus, of patients with major depression [1,2,3,4,5,6,7,8,9]. In particular, these studies revealed that the astroglial population was reduced and the size of astrocyte increased in the corticolimbic regions of individuals with major depression [1,2,3,10,11,12]. These findings indicated the possibility that a decrease in astrocyte population is compensated by glial cell enlargement in the pathophysiology/pathomechanisms of mood disorders [13]. This hypothesis is supported by several preclinical studies in which the expression of glial fibrillary acidic proteins decreased in several experimental depression rodent models [14,15]. Therefore, the dysfunctions of regulation systems of transmission and homeostasis in astrocytes probably play important roles in the pathophysiology/pathomechanisms of mood disorders [13]. Traditionally, tripartite synaptic transmission referred to transmissions of D-serine, L-glutamate and ATP between neurons and astrocytes, whereas the latest interpretation of tripartite synaptic transmission has been extended to other transmission systems, such as monoaminergic tripartite synaptic transmissions [16]. Indeed, the expression of several serotonin (5-HT) receptors, including 5-HT1AR and 5-HT7R, in the astrocytes has been demonstrated [17,18]. The concept of the participation of monoaminergic transmission in tripartite synaptic transmission provides an opportunity to expand the novel monoaminergic hypothesis in which the functional modulation of astrocytes is involved in a pathophysiological mechanism of a part of neuropsychiatric disorders [13,19].

Hemichannels and gap junctions are well known to modulate the network of astrocytes [20,21,22]. Connexin43 (Cx43) is the most widely and predominantly expressed molecule (connexin family), constituting hemichannels and gap junctions in the central nervous system, including astrocytes [23]. Hemichannels and gap junctions are constructed by respective single and dual connexons, which are assembled by six connexin units [24]. Dual connexons in two neighbouring cells (gap junction) with an aqueous pore and charged surface walls play important roles in homeostasis via intracellular pan-neuroglial networks [20,21]. A single connexon (hemichannel) contributes to tripartite synaptic transmission via a chemical connection between the intra and extracellular spaces [22]. The transmembrane pores of both hemichannels and gap junctions are permeable to transmitters, second messengers, mRNA, purines, signalling mediators and ions up to 1.5 kDa [24]. Astrocytes release gliotransmitters via exocytotic and non-exocytotic (reverse astroglial transporter and hemichannel) systems [20,21,22]. However, in the resting stage, astroglial gap junctions are functional in that they are permeable to various intracellular molecules, whereas astroglial hemichannels are unfunctional due to a low opening probability [25,26,27]. Contrary to the resting stage, the depolarization and attenuation of the concentration gradients between extracellular and intracellular levels of K^+^ and Ca^2+^ induced by pathological conditions, including epileptic discharge, hypoxia and ischemia, enhance the hemichannel opening probability (activation of hemichannel) [28,29]. Therefore, it has been established that gap junctions and hemichannels play fundamental roles in the physiological and pathological states, respectively [13,25]. Indeed, recent neuropsychopharmacological studies have suggested that the functional abnormalities of extracellular tripartite synaptic transmission and intracellular pan-neuroglial networks in several pathways, including the mesocortical and thalamocortical pathways, play important roles in the pathomechanisms and pathophysiology of various neuropsychiatric disorders, such as schizophrenia, epilepsy and mood disorders [13,25,30,31]. Our recent studies demonstrated that several mood-stabilising antiseizure drugs and mood-stabilising antipsychotics also affected gliotransmitter release via the enhancement of astroglial hemichannel activities and the expression of the Cx43 protein in the astroglial plasma membrane; however, careful consideration is required with respect to whether these effects contribute to clinical outcomes due to inconsistent results [13,31].

A recent study reported that the genetic inactivation of Cx43 enhanced the antidepressant action of acute fluoxetine administration [32]. However, on the other hand, the chronic administration of fluoxetine, fluvoxamine and duloxetine increased the frontal expression of mRNA and the Cx43 protein in vivo [33,34,35,36]. Behavioural studies have reported that experimental depression rodent models (corticosterone administration model, chronic unpredictable stress model and acute/chronic restraint stresses models) had a decreased expression of mRNA and Cx43 protein in the frontal cortex, whereas these depression models displayed a contradiction between suppressed gap junction permeability and enhanced hemichannel permeability [33,37,38,39,40,41]. Therefore, in depression models, transmissions via astroglial gap junctions and hemichannels are probably suppressed and enhanced, respectively [13]. This hypothesis has been supported by post-mortem studies on the reduction of mRNA and Cx43 protein expression in various brain regions of patients with depression and victims of suicide [5,6,7,8,9].

As mentioned above, a number of previous pharmacological studies suggested that the functional abnormalities of astroglial transmission associated with Cx43 contributed to the pathophysiology/pathomechanisms of mood disorders; however, the functional abnormality of Cx43 associated with mood disorders is more complicated than expected, as the findings regarding Cx43 expression and the functions of the abnormalities in depression models and the effects of the 5-HT transporter inhibiting antidepressants are inconsistent [33,34,35,36,37,38,39,40,41]. We hypothesised that excluding the influence of pan-neuroglial networks could show us the primary role of astroglial function without secondary astroglial roles via neuronal mechanisms, in order to identify the astroglial pathophysiology of depression. These complicated responses of astroglial Cx43 to 5-HT transporter inhibiting agents are possibly caused by non-selective activation of any 5-HT receptor subtypes due to increasing extracellular 5-HT levels. In other words, we speculate that an enhancement of broad-spectrum 5-HT receptor subtypes can ordinarily contribute to an antidepressant-like action, whereas it has not been clarified which 5-HT receptor subtypes contribute to the antidepressant-like action and/or which 5-HT receptor subtypes negatively affect the antidepressant-like action of the 5-HT transporter-inhibiting antidepressants [42]. Additionally, the fundamental targeting 5-HT receptor subtypes which increase synthesis and expression in the plasma membrane (trafficking) of the Cx43 protein also remained to be clarified. Another view is that astroglial glutamatergic transmission plays an important role in the pathophysiology of depression [43,44,45]. It is well known that the two major origins of glutamate are exhibited in astrocytes. Astrocytes synthesise glutamate from α-ketoglutarate via an amino-transfer reaction and a tricarboxylic acid cycle [46]. The other pathway is the uptake of released glutamate from neurones by glutamate transporters [47]. Indeed, an aconitase inhibitor, fluorocitrate, drastically supresses astroglial releases of L-glutamate and D-serine in vivo and in vitro [48,49]. Therefore, in accordance with these previous preclinical findings, to explore the pathophysiology of mood disorders associated with astrocytes, the present study determined the effects of escitalopram (selective 5-HT transporter inhibitor) and vortioxetine (5-HT partial agonist reuptake inhibitor: SPARI) [50,51] on astroglial L-glutamate release through activated hemichannels and Cx43 expression using primary cultured rat cortical astrocytes. Furthermore, to clarify the effects of 5-HT receptor subtypes on astroglial L-glutamate release through hemichannels and astroglial Cx43 dynamics, the present study also determined the effects of the selective 5-HT1AR agonist (BP554), antagonist (WAY100635) and the 5-HT7R inhibitor (SB269970) on astroglial L-glutamate release and expression of 5-HT1AR, 5-HT7R and Cx43 in the astroglial plasma membrane.

## 2. Results

### 2.1. Effects of Acute and Subchronic Administrations of Escitalopram and Vortioxetine on Astroglial L-Glutamate Release

Therapeutic-relevant serum concentrations of escitalopram and vortioxetine are 50~250 nM and 50~200 nM, respectively [52,53]. Based on these clinical data, in the present study, cortical primary cultured astrocytes were acutely (for 120 min) and subchronically (for seven days) administered with therapeutic-relevant and supratherapeutic concentrations of escitalopram and vortioxetine (50, 100, 200 and 400 nM). Prior to the determination of the levels of L-glutamate release from the cultured astrocytes, levels of 5-HT in an incubation medium and artificial cerebrospinal fluid (ACSF) could not be detected. Briefly, to study the concentration-dependent effects of escitalopram and vortioxetine on astroglial L-glutamate release, during 21–28 days after culture (DIV), cortical astrocytes were incubated in Dulbecco’s Modified Eagle Medium containing 10% foetal calf serum (fDMEM) without (control or acute administration) and with (subchronic administration) escitalopram and vortioxetine (50, 100, 200 and 400 nM) for seven days. At DIV28, after a washout with ACSF, cortical primary cultured astrocytes were incubated in ACSF with or without (control) escitalopram or vortioxetine for 120 min (acute or subchronic administrations: pre-treatment period). After the pre-treatment period, astrocytes were then incubated in 100 μL of ACSF containing the same agents of the pre-treatment (20 min), and the ACSF was collected for analysis. During the resting stage, astroglial hemichannels exhibit a low opening probability, whereas increased extracellular K^+^ with decreased extracellular Ca^2+^ activates astroglial hemichannel activity [54,55,56]. Therefore, according to previous studies [54,55,56], to determine the concentration-dependent effects of acute and subchronic administrations of escitalopram and vortioxetine on astroglial L-glutamate release through activated astroglial hemichannels, after the collection of ACSF as a basal release, the primary cultured astrocytes were incubated in high (100 mM) K^+^ with Ca^2+^-free ACSF (FCHK-ACSF) containing the same agent of the pre-treatment period for 20 min (FCHK-evoked stimulation). The FCHK-evoked release was calculated by subtracting the levels of basal release (in ACSF) from the levels of L-glutamate in FCHK-ACSF.

**Figure 1:**
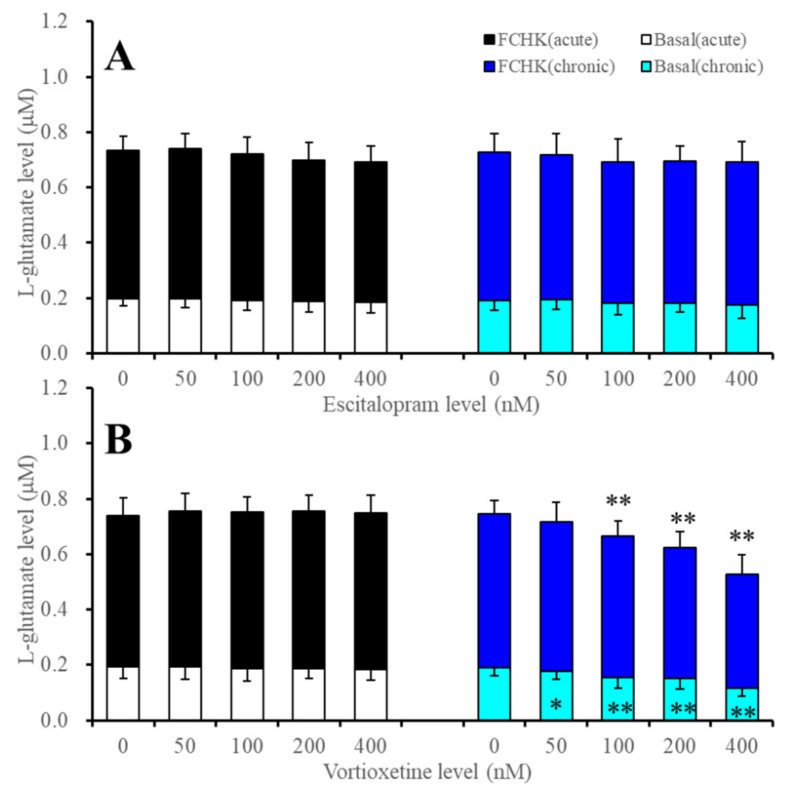
Concentration-dependent effects of the acute and subchronic administrations of (**A**) escitalopram (50, 100, 200 and 400 nM) and (**B**) vortioxetine (50, 100 200 and 400 nM) on the astroglial basal and high (100 mM) K^+^ with Ca^2+^ free (FCHK-ACSF) evoked L-glutamate releases. Ordinate: mean ± SD (*n* = 6) of the astroglial L-glutamate release (μM), and abscissa: concentration of escitalopram and vortioxetine (nM). * p < 0.05, ** p < 0.01: relative to antidepressant free by MANOVA with Tukey’s post hoc test. The FCHK-evoked release was calculated by subtracting the levels of L-glutamate in ACSF (opened and light blue columns) from the levels of L-glutamate in FCHK-ACSF (closed and blue columns).

Neither acute nor subchronic administrations of escitalopram (50, 100, 200 and 400 nM) affected basal or FCHK-evoked astroglial L-glutamate releases (F_stimulation_(1,10) = 1273.8 (*p* < 0.01), F_Administration × release_(1,10) = 0.1 (*p* > 0.1), F_Level_(2.6,25.6) = 2.9 (*p* > 0.05), F_Administration × level_(2.6,25.6) = 0.3 (*p* > 0.1), F_Stimulation × level_(4,40) = 0.4 (*p* > 0.1), F_Administration × stimulation × level_(4,40) = 0.4 (*p* > 0.1), F_Administration_(1,10) = 0.1 (*p* > 0.1)) (Figure 1A). Contrary to escitalo*p*ram, vortioxetine (50, 100, 200 and 400 nM) affected basal or FCHK-evoked astroglial L-glutamate releases (F_stimulation_(1,10) = 2972.4 (*p* < 0.01), F_Administration × release_(1,10) = 6.4 (*p* < 0.05), F_Level_(4,40) = 31.5 (*p* < 0.01), F_Administration × level_(4,40) = 35.7 (*p* < 0.01), F_Stimulation × level_(4,40) = 2.3 (*p* > 0.05), F_Administration × stimulation×level_(4,40) = 7.7 (*p* < 0.01), F_Administration_(1,10) = 3.2 (*p* > 0.1)) (Figure 1B). The acute administration of vortioxetine (50, 100, 200 and 400 nM) did not affect basal or FCHK-evoked astroglial L-glutamate releases, but subchronic vortioxetine administration (50, 100, 200 and 400 nM) concentration-dependently decreased basal and FCHK-evoked astroglial L-glutamate releases (Figure 1B).

The discrepant effects between escitalopram and vortioxetine on astroglial L-glutamate release from cultured astrocytes suggest that acute and subchronic administrations of escitalopram and acute administration of vortioxetine do not affect astroglial L-glutamate release, but subchronic administration of vortioxetine directly suppresses astroglial L-glutamate release, since the 5-HT levels in the incubation medium (fDMEM) and in ACSF were lower than 0.1 nM.

### 2.2. Effects of Elective 5-HT Receptor Agents on Astroglial L-Glutamate Release

Vortioxetine exhibits an affinity with rat 5-HT transporter (Ki = 8.6 nM), 5-HT7R (Ki = 200 nM) and 5-HT1AR (Ki = 230 nM) [19,57,58]. The expression of 5-HT1AR and 5-HT7R in astrocytes has been established [17,18,19]. Therefore, to explore the mechanisms of the inhibitory effects of subchronic administration of vortioxetine on astroglial glutamatergic transmission, the present study determined the effects of 5-HT1AR and 5-HT7R on astroglial L-glutamate release. Cortical astrocytes were incubated in fDMEM without (control or acute administration) and with (subchronic administration) 5-HT receptor agents for seven days. At DIV28, after a washout with ACSF, cultured astrocytes were incubated in ACSF with or without (control) the 5-HT receptor agent (pre-treatment). After the pre-treatment period, astrocytes were then incubated in 100 μL ACSF or FCHK-ACSF containing the same agents as the pre-treatment (20 min).

#### 2.2.1. Effects of Acute and Subchronic Administration of 5-HT1AR and 5-HT7R Agents on Astroglial L-Glutamate Release during Resting Stage and through Activated Hemichannels (Study 3)

To clarify the mechanisms of the inhibitory effects of the subchronic administration of vortioxetine associated with 5-HT1AR and 5-HR7R on both basal and FCHK-evoked astroglial L-glutamate releases, the acute and subchronic administration of 5-HT1AR agonist, BP554 (50 μM), 5-HT1AR antagonist, WAY100635 (10 μM), and 5-HT7R inhibitor, SB269970 (10 μM), on astroglial L-glutamate release were determined [50,59,60].

**Figure 2:**
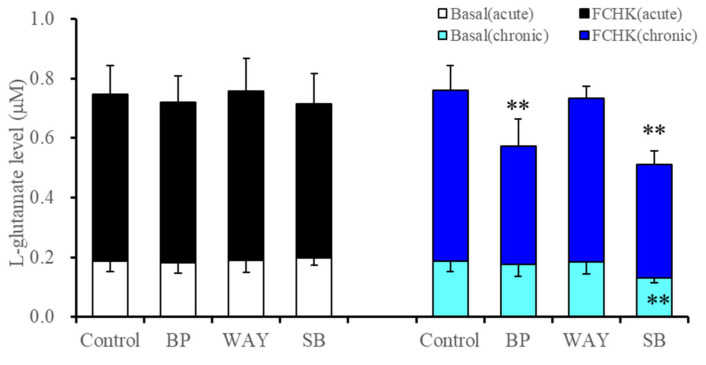
Effects of acute and subchronic administrations of selective 5-HT receptor agents, 5-HT1AR agonist, BP554 (BP: 50 μM), 5-HT1AR antagonist WAY100635 (WAY: 10 μM) and 5-HT7R inhibitor, SB269970 (SB: 10 μM) on basal and FCHK-evoked astroglial L-glutamate releases. Ordinate: mean ± SD (*n* = 6) of astroglial L-glutamate release (μM). ** *p* < 0.01: relative to control (without any 5-HT receptor agents) by two-way analysis of variance (ANOVA) with Tukey’s post hoc test. The FCHK-evoked release was calculated by subtracting the levels of L-glutamate in ACSF (opened and light blue columns) from the levels of L-glutamate in FCHK-ACSF (closed and blue columns).

Neither acute nor subchronic administrations of BP554 or WAY100635 affected basal astroglial L-glutamate release (Figure 2). Acute administration of SB269970 also did not affect basal astroglial L-glutamate release, but the subchronic administration of SB269970 decreased basal astroglial L-glutamate release (F_SB269970_(1,20) = 3.8 (*p* > 0.05), F_During_(1,20) = 7.7 (*p* < 0.05), F_SB269970*During_(1,20) = 8.6(*p* < 0.01)) (Figure 2). Neither acute nor subchronic administrations of WAY100635 affected FCHK-evoked astroglial L-glutamate release (Figure 2). Acute administration of BP554 and SB269970 also did not affect FCHK-evoked astroglial L-glutamate release, but the subchronic administration of BP554 (F_BP554_(1,20) = 7.5 (*p* < 0.05), F_During_(1,20) = 3.4 (*p* > 0.05), F_BP554*During_(1,20) = 4.6 (*p* < 0.05)) and SB269970 (F_SB269970_(1,20) = 12.2 (*p* < 0.01), F_During_(1,20) = 3.5 (*p* > 0.05), F_SB269970*During_(1,20) = 4.8 (*p* < 0.05)) decreased FCHK-evoked astroglial L-glutamate release (Figure 2).

These results indicate that astroglial L-glutamate release is, at least partially, regulated by both 5-HT1AR and 5-HT7R, possibly via the expression of the hemichannel constitutive protein, as both BP554 and SB269970 acutely did not affect astroglial release, but their subchronic administration decreased astroglial L-glutamate release.

#### 2.2.2. Interaction between Vortioxetine and 5-HT1AR Antagonist (WAY100635) on Basal Astroglial L-Glutamate Release and through Activated Hemichannels

To clarify the effects of the 5-HT1AR agonistic action of vortioxetine on astroglial transmission [19], the interaction between therapeutic-relevant concentrations of vortioxetine (200 nM) and the 5-HT1AR antagonist, WAY100635 (10 μM), on basal and FCHK-evoked astroglial L-glutamate release were determined.

**Figure 3:**
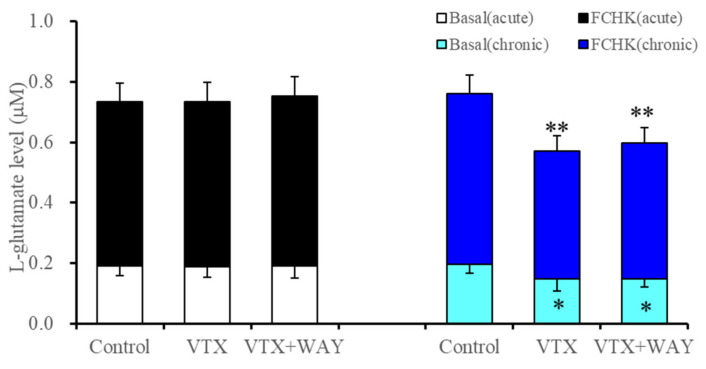
Interaction between acute and subchronic administrations of therapeutic-relevant concentration of vortioxetine (200 nM: VTX) and vortioxetine (200 nM) plus WAY100635 (5-HT1AR antagonist: 10 μM) (VTX+WAY) on basal and FCHK-evoked astroglial L-glutamate release. Ordinate: mean ± SD (*n* = 6) of astroglial L-glutamate release (μM). * *p* < 0.05, ** *p* < 0.01: relative to control by one-way ANOVA with Tukey’s post hoc test. The FCHK-evoked release was calculated by subtracting the levels of L-glutamate in ACSF (opened and light blue columns) from the levels of L-glutamate in FCHK-ACSF (closed and blue columns).

Acute administration of vortioxetine did not affect basal and FCHK-evoked astroglial L-glutamate release (Figure 3). The subchronic administration of vortioxetine decreased basal astroglial L-glutamate release, whereas the inhibitory effects of subchronic vortioxetine administration were not affected by the acute administration of WAY100635 (F(2,15) = 4.6 (*p* < 0.05)) (Figure 3). Similar to basal release, the subchronic administration of vortioxetine decreased FCHK-evoked astroglial L-glutamate release, whereas the inhibitory effects of subchronic vortioxetine administration were not affected by the subchronic administration of WAY100635 (F(2,15) = 11.0 (*p* < 0.01)) (Figure 3). The results in Study 4 suggest the possibility that the 5-HT1AR agonistic effects of vortioxetine cannot provide the inhibitory effects of the subchronic administration of vortioxetine on astroglial glutamatergic transmission associated with hemichannels.

### 2.3. Effects of Subchronic Administration of 5-HT Receptor Agents, Therapeutic-Relevant Concentrations of Escitalopram and Vortioxetine, on Expression of Proteins Associated with Astroglial L-Glutamate Release

#### 2.3.1. Effects of Subchronic Administration of 5-HT Receptor Agents, Therapeutic-Relevant Concentrations of Escitalopram and Vortioxetine, on Cx43 Expression in the Cytosol and Plasma Membrane Fractions of Primary Cultured Astrocytes (Study 5)

**Figure 4:**
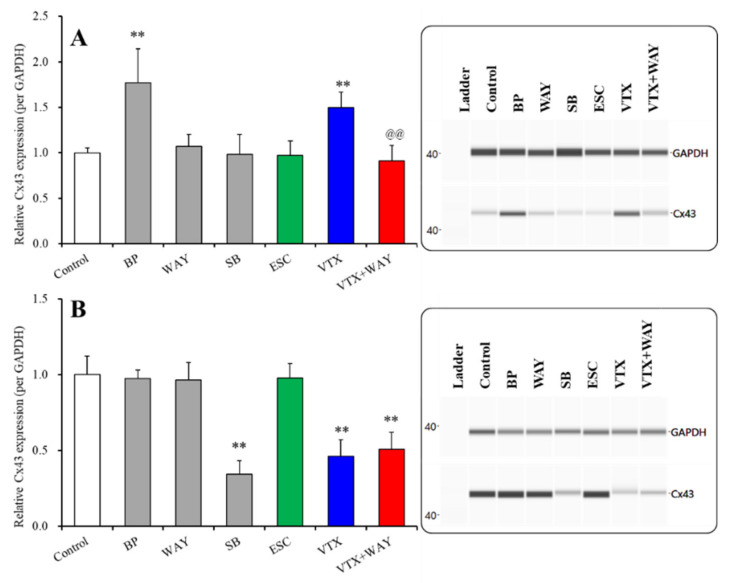
Effects of subchronic administration of 5-HT receptor agents, 5-HT1AR agonist, BP554 (BP: 50 μM), 5-HT1AR antagonist, WAY100635 (WAY: 10 μM), 5-HT7R inhibitor, SB269970 (SB: 10 μM), therapeutic-relevant concentration of escitalopram (ESC: 200 nM) and vortioxetine (VTX: 200 nM) on Cx43 protein expression in the cytosol (**A**) and plasma membrane (**B**) fractions. In left side histograms, ordinate: mean ± SD (*n* = 6) of the relative protein level of Cx43 per glyceraldehyde 3-phosphate dehydrogenase (GAPDH). ** p < 0.01: relative to control by student *t*-test or one-way ANOVA with Tukey’s post hoc test. ^@@^ *p* < 0.01: relative to vortioxetine alone (VTX) by one-way ANOVA with Tukey’s post hoc test. Right side panels indicate their pseudo-gel images, using capillary immunoblotting.

The subchronic administration of 50 μM of BP554 (5-HT1AR agonist) increased Cx43 expression in the cytosol fraction (Figure 4), but did not affect Cx43 expression in the plasma membrane fraction (Figure 4). Neither acute nor subchronic administrations of 10 μM of WAY100635 (5-HT1AR antagonist) affected Cx43 expression in the cytosol and plasma membrane fractions (Figure 4). Contrary to 5-HT1AR, the subchronic administration of 10 μM of SB269970 (5-HT7R inhibitor) decreased Cx43 expression in the plasma membrane fraction without affecting Cx43 expression in the cytosol fraction (Figure 4). Therefore, the activation of astroglial 5-HT1AR probably enhances Cx43 synthesis, but does not affect Cx43 trafficking to the plasma membrane in the astrocytes; whereas, conversely, the inhibition of 5-HT7R probably suppresses Cx43 trafficking to the plasma membrane without affecting Cx43 synthesis.

The subchronic administration of a therapeutic-relevant concentration of escitalopram (200 nM) did not affect Cx43 expression in the cytosol and plasma membrane fractions (Figure 4); however, the subchronic administration of a therapeutic-relevant concentration of vortioxetine (200 nM) increased and decreased Cx43 expression in the cytosol and plasma membrane fractions, respectively (Figure 4). The inhibition of 5-HT1AR prevented the stimulatory effects of vortioxetine on Cx43 protein expression in the cytosol (F(2,15) = 30.9 (*p* < 0.01)) (Figure 4), whereas the inhibitory effects of vortioxetine on Cx43 protein expression in the plasma membrane was not affected by the 5-HT1AR antagonist (F(2,15) = 40.5 (*p* < 0.01)) (Figure 4).

Therefore, the opposite effects of the subchronic administration of therapeutic-relevant concentrations of vortioxetine on Cx43 protein expression in the increased cytosol and decreased plasma membrane fractions are probably generated by the activation of 5-HT1AR and the inhibition of 5-HT7R, respectively. In other words, vortioxetine probably enhances Cx43 synthesis, but suppresses Cx43 trafficking to the plasma membrane.

#### 2.3.2. Effects of Subchronic Administrations of 5-HT Receptor Agents, Therapeutic-Relevant Concentrations of Escitalopram and Vortioxetine, on Expression of 5-HT1AR and 5-HT7R in the Plasma Membrane Fractions of Primary Cultured Astrocytes

It is well known that the chronic administration of selective 5-HT transporter inhibitors and 5-HT1AR partial agonists leads to the downregulation or desensitization of 5-HT1AR. These reduced inhibitory functions of 5-HT1AR generate the enhancement of serotonergic transmission [61]. Contrastingly, the chronic administration of 5-HT7R inhibiting agents leads to the downregulation of 5-HT7R resulting in an attenuation of serotonergic transmission [50,51,59,60]. Taken together with these previous preclinical findings, the results in Study 5 are modulated by the downregulation or desensitization of 5-HT receptors. Therefore, to explore the more detailed mechanisms of the increased expression in the cytosol and the decreased expression in the plasma membrane of Cx43 protein induced by subchronic vortioxetine administration, the effects of the subchronic administration of therapeutic-relevant concentrations of vortioxetine on 5-HT1AR and 5-HT7R in the plasma membrane fraction were determined.

**Figure 5:**
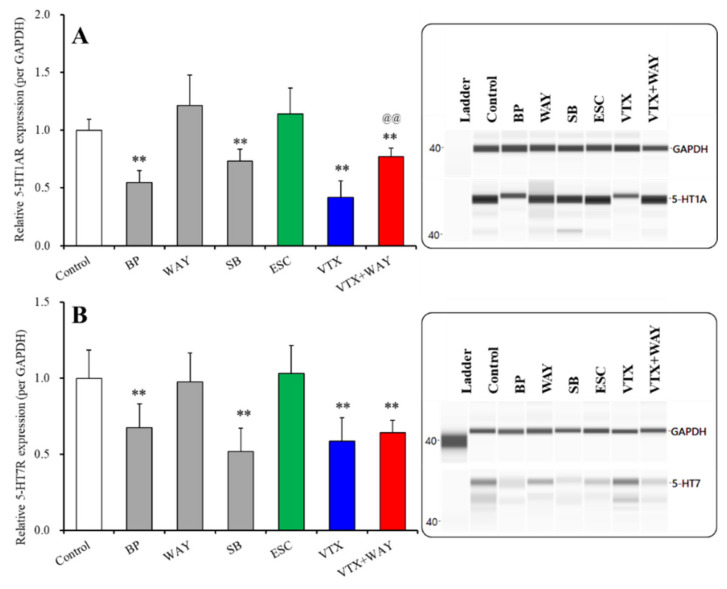
Effects of subchronic administration of 5-HT receptor agents, 5-HT1AR agonist, BP554 (BP: 50 μM), 5-HT1AR antagonist, WAY100635 (WAY: 10 μM), 5-HT7R antagonist, SB269970 (SB: 10 μM), therapeutic-relevant concentration of escitalopram (ESC: 200 nM) and vortioxetine (VTX: 200 nM) on 5-HT1AR (**A**) and 5-HT7R (**B**) protein expression in the plasma membrane fractions. In left side histograms, ordinate: mean ± SD (*n* = 6) of the relative protein level of 5-HT1AR and 5-HT7R per GAPDH. ** p < 0.01: relative to control by student *t*-test or one-way ANOVA with Tukey’s post hoc test. ^@@^ *p* < 0.01: relative to vortioxetine alone (VTX) by one-way ANOVA with Tukey’s post hoc test. Right side panels indicate their pseudo-gel images, using capillary immunoblotting.

The subchronic administration of 50 μM of BP554 (5-HT1AR agonist) and 10 μM of SB269970 (5-HT7R antagonist) decreased 5-HT1AR expression in the plasma membrane fraction, whereas 10 μM of WAY100635 (5-HT1AR antagonist) did not affect 5-HT1AR expression in the plasma membrane fraction (Figure 5A). The subchronic administration of a therapeutic-relevant concentration of escitalopram (200 nM) did not affect 5-HT1AR expression in the plasma membrane fraction (Figure 5A). In contrast, the subchronic administration of a therapeutic-relevant concentration of vortioxetine (200 nM) decreased 5-HT1AR expression in the plasma membrane fraction (Figure 5A). The subchronic administration of the 5-HT1AR antagonist, WAY100635 (10 μM), prevented the inhibitory effects of vortioxetine on 5-HT1AR expression in the plasma membrane fraction (F(2,15) = 21.8 (*p* < 0.01)) (Figure 5A). These results indicate that the subchronic activation of 5-HT1AR and the inhibition of 5-HT7R in astrocytes suppress/downregulate 5-HT1AR expression in the plasma membrane of astrocytes.

Similar to 5-HT1AR, the subchronic administration of 50 μM of BP554 and 10 μM of SB269970 decreased 5-HT7R expression in the plasma membrane fraction (Figure 5B). WAY100635 (10 μM) did not affect 5-HT7R expression in the plasma membrane fraction (Figure 5B). The subchronic administration of a therapeutic-relevant concentration of escitalopram (200 nM) did not affect 5-HT7R expression in the plasma membrane fraction, whereas the subchronic administration of a therapeutic-relevant concentration of vortioxetine (200 nM) decreased 5-HT7R expression in the plasma membrane fraction (Figure 5B). The subchronic administration of WAY100635 (10 μM) did not affect the inhibitory effects of vortioxetine on 5-HT7R expression in the plasma membrane fraction (F(2,15) = 13.9 (*p* < 0.01)) (Figure 5B). These results indicate that the subchronic activation of 5-HT1AR and the inhibition of 5-HT7R in astrocytes suppress/downregulate 5-HT7R expression in the plasma membrane of astrocytes.

Therefore, the subchronic administration of a therapeutic-relevant concentration of vortioxetine downregulates both 5-HT1AR and 5-HT7R. However, the downregulations of 5-HT1AR and 5-HT7R were predominantly produced by the activation of 5-HT1AR and the inhibition of 5-HT7R, respectively, since WAY100635 inhibited 5-HT1AR downregulation without affecting 5-HT7R downregulation.

#### 2.3.3. Effects of Subchronic Administrations of 5-HT Receptor Agents, Therapeutic-Relevant Concentrations of Escitalopram and Vortioxetine, on Phosphorylation of Extracellular Signal-Regulated Kinase (ERK) in the Plasma Membrane Fractions of Primary Cultured Astrocytes

The results in Study 5 suggest that the subchronic administration of a therapeutic-relevant concentration of vortioxetine enhances Cx43 synthesis but suppresses Cx43 trafficking to the plasma membrane. The post-translational modification of Cx43, including the phosphorylation, acetylation, nitrosylation, sumoylation and ubiquitylation, contribute to the trafficking of Cx43 to the plasma membrane. The phosphorylation of Cx43 was found to be regulated by the action of more than 10 kinases and phosphatases, including mitogen-activated protein kinase/extracellular signal-regulated kinase (ERK) signalling [13,25,31,62]. Both 5-HT1AR and 5-HT7R affect ERK signalling in serotonergic neurons [19,63]. Based on these previous findings, to clarify the mechanisms of the inhibitory effects of vortioxetine on astroglial Cx43 trafficking to the plasma membrane in astrocytes, the effects of the subchronic administration of a therapeutic-relevant concentration of vortioxetine on the expression of ERK and phosphorylated ERK in the plasma membrane fraction were determined.

**Figure 6:**
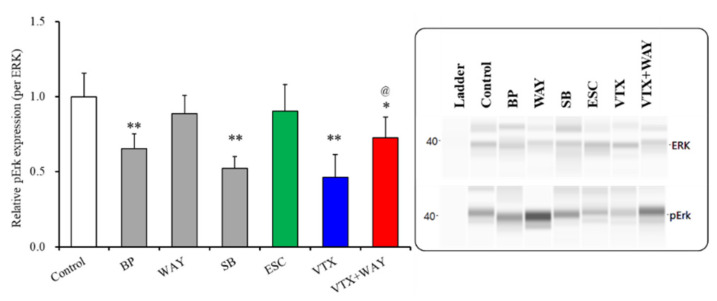
Effects of subchronic administration of serotonin receptor agents, 5-HT1AR agonist. BP554 (BP: 50 μM), 5-HT1AR antagonist, WAY100635 (WAY: 10 μM), 5-HT7R antagonist, SB269970 (SB: 10 μM), therapeutic-relevant concentration of escitalopram (ESC: 200 nM) and vortioxetine (VTX: 200 nM) on phosphorylated Erk (pErk) level in the plasma membrane fractions. In left side histograms, ordinate: mean ± SD (*n* = 6) of the relative protein level of pErk per ERK. * p < 0.05, ** p < 0.01: relative to control by student T-test or one-way ANOVA with Tukey’s post hoc test. *^@^ p* < 0.05: relative to vortioxetine alone (VTX) by one-way ANOVA with Tukey’s post hoc test. Right side panels indicate their pseudo-gel images, using capillary immunoblotting.

The subchronic administration of both 50 μM BP554 (5-HT1AR agonist) and 10 μM SB269970 (5-HT7R inhibitor) decreased phosphorylated ERK in the plasma membrane fraction, whereas 10 μM of WAY100635 (5-HT1AR antagonist) did not affect phosphorylated ERK in the plasma membrane fraction (Figure 6). The subchronic administration of a therapeutic-relevant concentration of escitalopram (200 nM) did not affect phosphorylated ERK, whereas that of vortioxetine (200 nM) decreased phosphorylated ERK in the plasma membrane fraction (Figure 6). The subchronic administration of 5-HT1AR antagonist, WAY100635 (10 μM), antagonized the inhibitory effects of vortioxetine on phosphorylated ERK in the plasma membrane fraction, but the level of phosphorylated ERK was lower compared with that of the control (F(2,15) = 23.0 (*p* < 0.01)) (Figure 6). These results (5-HT1AR agonist (BP554) and antagonist (WAY100635) reduced and had no effect on phosphorylated ERK, respectively) suggest that 5-HT1AR phasically suppresses the phosphorylation of ERK.

## 3. Discussion

### 3.1. Effects of 5-HT Receptors on Astroglial L-Glutamate Release and Protein Expression Associated with Its Regulation Mechanisms in Astrocytes

The present study demonstrated that the subchronic administration of the 5-HT1AR agonist (BP554), the 5-HT7R inhibitor (SB269970) and a therapeutic-relevant concentration of vortioxetine, at least partially, affected astroglial L-glutamate release or protein expressions of Cx43, 5-HT1AR and 5-HT7R, whereas neither the subchronic administration of 5-HT1AR antagonist (WAY100635) nor a therapeutic-relevant concentration of escitalopram affected them (Table 1). In the present study, astrocytes were subchronically incubated in a 5-HT-free environment. Therefore, it is easy to interpret that the selective 5-HT transporter inhibitor, escitalopram, and the 5-HT1AR antagonist, WAY100635, have no effect on the astroglial transmission associated with 5-HT in the 5-HT-free environment. In contrast to escitalopram and WAY100635, although SB269970 is considered to be a selective 5-HT7R inhibitor, in a 5-HT-free environment, SB269970 displays pharmacological effects on the astroglial transmission associated with the serotonergic system. This contradictive demonstration of SB269970 suggests that SB269970 may be a 5-HT7R inverse agonist, rather than a 5-HT7R antagonist. Indeed, several previous studies indicated the possibility that SB269970 was a 5-HT7R inverse agonist, as the chronic administration of SB269970 downregulated or desensitised 5-HT7R [50,64,65]. Therefore, in this report, we progress the consideration of SB269970 as a 5-HT7R inverse agonist. The demonstrated results in this study are summarised in Table 1.

Neither the acute administrations of BP554 nor SB269970 affected basal and FCHK-evoked astroglial L-glutamate release. In contrast to acute administration, the subchronic administration of BP554 suppressed FCHK-evoked astroglial L-glutamate release without affecting basal release, whereas the subchronic administration of SB269970 supressed both astroglial basal and FCHK-evoked L-glutamate releases. These results suggest that SB269970 (5-HT7R inverse agonist) does not directly inhibit astroglial hemichannel permeability, but possibly suppresses the expression of functional hemichannels in the astroglial plasma membrane.

It has been established that there are different neuronal 5-HT1AR signalling between the autoreceptor in serotonergic neurons and the heteroreceptor in non-serotonergic neurons [66,67]. The autoreceptor preferentially binds to Gi3, but the heteroreceptor binds to Gαi2 or Gαo [66,67]. The activation of a 5-HT1AR autoreceptor by a selective 5-HT transporter inhibitor generates the desensitisation or downregulation of the 5-HT1AR autoreceptor that requires weeks of treatment; however, the 5-HT1AR heteroreceptor is resistant to 5-HT transporter inhibitor-induced desensitisation and downregulation [68,69]. The 5-HT1AR autoreceptor in the raphe nucleus suppresses ERK phosphorylation [70], whereas the 5-HT1AR heteroreceptor in the hippocampus conversely increases ERK phosphorylation [71]. In the present study, the subchronic activation of astroglial 5-HT1AR by BP554 decreased phosphorylated ERK and downregulated 5-HT1AR in the astroglial plasma membrane. Therefore, the 5-HT1AR that is expressed in the astroglial plasma membrane is possibly linked to signalling transduction systems resembling the 5-HT1AR autoreceptor. Furthermore, the subchronic administration of BP554 also downregulated 5-HT7R in the astroglial plasma membrane. The activation of the 5-HT1AR autoreceptor inhibited adenylate cyclase, whereas 8-OH-DPAT could not inhibit adenylyl cyclase [67], however buspirone was inhibited [72]. It is well known that 8-OH-DPAT is an agonist of both 5-HT1AR and 5-HT7R [73]. Therefore, these differences in agent dependence (between 8-OH-DPAT and buspirone) on intracellular signalling may be modulated by the intervention of the signalling of 5-HT7R.

Similar to BP554, under 5-HT-free conditions, the subchronic inhibition of 5-HT7R by SB269970 decreased the expression of 5-HT1AR, 5-HT7R and phosphorylated ERK in the astroglial plasma membrane. A number of functional 5-HT7R inhibitors, including SB269970, clozapine, olanzapine, lurasidone and vortioxetine, chronically downregulated the expression of 5-HT7R [50,65]. These demonstrations suggest that these 5-HT7R inhibitors are considered to be 5-HT7R inverse agonists rather than 5-HT7R antagonists [50,65]. The 5-HT7R family is composed of four functional splice variants in rodents (5-HT7Ra, 5-HT7Rb and 5-HT7Rc) and in humans (5-HT7Ra, 5-HT7Rb and 5-HT7d) [19]. Functional differences among the 5-HT7R splice variants have shown that 5-HT7Ra only activates Gs-independent types 1 and 8 of adenylyl cyclase, resulting in enhanced ERK signalling [19]. Indeed, SB269970 suppresses adenylyl cyclase activities [65,74]. Additionally, the present study demonstrated that subchronic SB269970 administration reduced phosphorylated ERK in the astroglial plasma membrane.

Interestingly, the subchronic administration of both BP554 and SB269970 downregulated phosphorylated ERK, 5-HT1AR and 5-HT7R in the astroglial plasma membrane; however, the present study demonstrated the distinct effects between BP554 and SB269970 on Cx43 protein expression. The subchronic administration of SB269970 suppressed Cx43 expression in the plasma membrane without affecting Cx43 expression in the cytosol; however, conversely, the subchronic administration of BP554 increased Cx43 expression in the cytosol without affecting Cx43 expression in the plasma membrane. The trafficking of Cx43 to the plasma membrane is regulated by various post-transcriptional processes, acetylation, nitrosylation, sumoylation, ubiquitylation and phosphorylation, including ERK signalling [13,25,54,62,75]. Therefore, it is easy to interpret that subchronic administrations of SB269970 (5-HT7R inverse agonist) suppress Cx43 trafficking to the plasma membrane due to the inhibition of ERK phosphorylation, resulting in a reduction in astroglial L-glutamate release through hemichannels. In contrast to 5-HT7R, careful consideration is needed to interpret the effects of BP554 (5-HT1AR agonist), as the present study indicated two contradictive results. Subchronic BP554 administration increased the concentration of Cx43 in the cytosol fraction, but it was not affected in the plasma membrane fraction. Additionally, after subchronic exposure to BP554, although there were no effects in the expression of Cx43 in the plasma membrane, astroglial L-glutamate release through activated hemichannels was reduced. The discrepancy between Cx43 levels in the cytosol and the plasma membrane fractions is possibly explained by the attenuation of ERK signalling induced by subchronic BP554. The other discrepancy between Cx43 expression in the plasma membrane and L-glutamate release through activated astroglial hemichannels suggests the possibility that subchronic 5-HT1AR activation suppresses hemichannel activity. Cx43 has various phosphorylation sites which receive inhibitory and excitatory regulation [76,77,78]. Taken together with the hemichannel regulation system associated with phosphorylation, the suppression of ERK phosphorylation induced by the subchronic administration of BP554 suggests that astroglial L-glutamate release through activated hemichannels is probably reduced by the attenuation of hemichannel activity. Further experiments are needed to clarify the mechanism of these contradictions.

In addition, the present study cannot explain the detailed mechanisms of the Cx43 dynamics contradiction between the increased Cx43 expression in the cytosol and the unchanged Cx43 expression in the plasma membrane through subchronic BP554 administration; however, we should discuss one mechanism candidate. Although not revealed in the central nervous system, the enhancement of adenosine monophosphate-activated protein kinase (AMPK) in the bladder was demonstrated to suppress Cx43 synthesis [79]. The enhancement of adenylyl cyclase activity suppresses the activities of several AMPK subfamilies [80]. These previous findings show us a candidate mechanism in that the downregulation of 5-HT1AR possibly upregulates Cx43 synthesis due to the inhibition of AMPK induced by the disinhibition of adenylyl cyclase. In other words, the 5-HT1AR-induced upregulation of Cx43 synthesis is not generated by the direct action of 5-HT1AR, but is rather a secondary disinhibition induced by 5-HT1AR downregulation. Future investigation will clarify this possible mechanism.

### 3.2. Effects of Vortioxetine on Astroglial L-Glutamate Release and Protein Expression Associated with the Astroglial Serotonergic System

The fewer effects of the subchronic administration of the selective 5-HT transporter inhibitor, escitalopram, on the expression of 5-HT1AR, 5-HT7R, Cx43 and phosphorylated ERK, under 5-HT-free conditions, confirm that the fundamental pharmacological mechanisms of escitalopram are generated by the modulation of several 5-HT receptor isoforms via the inhibition of the 5-HT transporter. Therefore, the effects of the subchronic administration of a therapeutic-relevant concentration of vortioxetine on astroglial L-glutamate release and Cx43 expression in the astroglial plasma membrane is modulated by its 5-HT receptor-binding profile but not by 5-HT transporter inhibition. Vortioxetine exhibits an antagonistic affinity to rat 5-HT3 receptors (Ki = 1.1 nM), 5-HT transporters (Ki = 8.6 nM) and 5-HT7R (Ki = 200 nM), and an agonistic affinity to rat 5-HT1AR (Ki = 230 nM) [19,57,58]. Although the affinity of vortioxetine to rat 5-HT7R and 5-HT1AR is relatively lower than that to rat 5-HT3R, our previous study demonstrated that the subchronic systemic administration of effective dose of vortioxetine (2.5 mg/kg/day) downregulated 5-HT7R in the thalamic plasma membrane [50]. Therefore, in the present study, a therapeutic-relevant concentration of vortioxetine (200 nM) performed its inverse agonistic property on rat astroglial 5-HT7R.

The effects of the subchronic administration of vortioxetine on astroglial L-glutamate release were similar to those of SB269970, since vortioxetine subchronically inhibited both basal and FCHK-evoked astroglial L-glutamate release without acutely affecting them (Table 1). These inhibitory effects of vortioxetine on astroglial L-glutamate release seemed not to be related to 5-HT1AR, since the 5-HT1AR antagonist (WAY100635) could not antagonise the inhibitory effects of vortioxetine on astroglial L-glutamate release (Table 1). These results suggest that the inhibitory effects of vortioxetine on astroglial basal and FCHK-evoked L-glutamate releases are regulated predominantly by 5-HT7R rather than 5-HT1AR. Furthermore, the vortioxetine-induced suppression of astroglial L-glutamate release is probably also modulated by the Cx43 expression level in the plasma membrane, since these suppressions were generated by the subchronic administration of vortioxetine, but not by acute administration. Indeed, the subchronic administration of therapeutic-relevant concentrations of vortioxetine decreased Cx43 expression in the plasma membrane fraction, but conversely increased Cx43 expression in the cytosol fraction. These opposite effects of the subchronic administration of vortioxetine on Cx43 expression between the cytosol and the plasma membrane were converted by the 5-HT1AR antagonist (WAY100635), since WAY100635 inhibited the stimulatory effects of vortioxetine on Cx43 expression in the cytosol fraction without affecting Cx43 expression in the plasma membrane fraction. These results exhibit the possibilities that the subchronic activation of 5-HT1AR increases Cx43 expression in the cytosol, and the subchronic suppression 5-HT7R decreases Cx43 expression in the plasma membrane.

The lack of effect of the acute administration of vortioxetine, and the fact that only subchronic administration of vortioxetine affected astroglial L-glutamate release and Cx43 expression, represents that the sustained activation of 5-HT1AR and the inhibition of 5-HT7R probably play important roles in these vortioxetine-induced changes in the astroglial transmission systems. In other words, the combination of the downregulations of 5-HT1AR with 5-HT7R is possibly a fundamental mechanism of the increased Cx43 expression in the cytosol and the reduced Cx43 expression in the plasma membrane. Therefore, the subchronic administration of therapeutic-relevant concentrations of vortioxetine probably increased Cx43 synthesis via 5-HT1AR downregulation, but decreased functional Cx43 expression via the suppression of Cx43 trafficking to the plasma membrane.

The contribution of the functional abnormality of gap junctions to the pathophysiology of depression has been clarified in detail in comparison with the functional abnormality of hemichannels [13,30]. Chronic unpredictable stress, experimental depression models and corticosterone suppress gap junction permeability [33,39,40,78], whereas other experimental depression models and acute/chronic restraint stress enhance hemichannel permeability [37]. These behavioural studies suggest that the suppression of homeostatic intracellular pan-neuroglial networks associated with gap junctions and/or the enhancement of extracellular tripartite synaptic transmission associated with hemichannels contribute to the pathomechanisms of depressive mood [13]. In contrast to the pathomechanisms, several 5-HT transporter inhibiting antidepressants, fluoxetine, paroxetine, venlafaxine and duloxetine, augment and inhibit the permeabilities of gap junctions and hemichannels, respectively [33,81]. Therefore, the inhibitory effects of vortioxetine on astroglial hemichannel activity demonstrated in this study are consistent with the pathophysiological hypothesis of depression associated with astroglial hemichannels thus far. To clarify the more detailed antidepressive mechanisms of vortioxetine, we shall report the effects of vortioxetine on astroglial gap junction activity and the effects of astroglial L-glutamate release on monoaminergic transmission in a further study. Especially, to identify the effects of vortioxetine on differentiated functions of gap junctions and hemichannels, we shall also determine the effects of vortioxetine on the uptake of selective dyes, Lucifer yellow (being selectively permeable to gap junctions) and ethidium bromide (being selectively uptaken by hemichannels) [82].

## 4. Materials and Methods

All animal care and experimental procedures described in this report complied with the Ethical Guidelines established by the Institutional Animal Care and Use Committee at Mie University, Tsu, Japan (No. 2019-3-R2, 24 May 2019) and are reported in accordance with the Animal Research: Reporting of In Vivo Experiments (ARRIVE) guidelines. Astrocytes were prepared using a protocol adapted from previously described methods [26,27,49,62,83,84,85].

### 4.1. Preparation of Primary Astrocyte Culture

Pregnant Sprague Dawley rats (SLC, Sizuoka, Japan) were housed individually in cages and kept in air-conditioned rooms (temperature, 22 ± 2 °C) set at 12 h light/dark cycle, with free access to food and water. Cultured astrocytes were prepared from cortical astrocyte cultures of neonatal Sprague Dawley rats (*n* = 42) sacrificed by decapitation at 0–24 h of age. The cerebral hemispheres were removed under dissecting microscope. Tissue was chopped into fine pieces using scissors and then triturated briefly with micropipette. Suspension was filtered using 70 µm nylon mesh (BD, Franklin Lakes, NJ, USA) and centrifuged. Pellets were then resuspended in 10 mL Dulbecco’s modified Eagle’s medium (D6546: Sigma-Aldrich, St. Louis, MO, USA) containing 10% foetal calf serum (fDMEM), which was repeated three times.

After culture for 14 days (DIV14), contaminating cells were removed by shaking in standard incubator for 16 h at 200 rpm. On DIV21, astrocytes were removed from flasks by trypsinization and seeded directly onto translucent poly ethylene terephthalate (PET) membrane (1.0 μm) with 24-well plates (BD) at a density of 100 cells/cm^2^ for experiments from DIV21 to DIV28, the culture medium (fDMEM) was changed twice a week, and escitalopram (50, 100, 200 and 400 nM), vortioxetine (50, 100, 200 and 400 nM), 5-HT1AR agonist, BP554 (50 μM), 5-HT1AR antagonist, WAY100635 (10 μM) and 5-HT7R inverse agonist, SB269970 (10 μM) were added for subchronic administrations (7 days). On DIV28, cultured astrocytes were washed out using artificial cerebrospinal fluid (ACSF), and this was repeated three times.

The ACSF was comprised of NaCl 150.0 mM, KCl 3.0 mM, CaCl_2_ 1.4 mM, MgCl_2_ 0.8 mM, and glucose 5.5 mM, buffered to pH 7.3 with 20 mM HEPES buffer. After the washout, astrocytes were incubated in ACSF (100 μL translucent PET membrane) containing escitalopram (50, 100, 200 and 400 nM), vortioxetine (50, 100, 200 and 400 nM), 5-HT1AR agonist, BP554 (50 μM), 5-HT1AR antagonist, WAY100635 (10 μM) and 5-HT7R antagonist, SB269970 (10 μM) at 35 °C for 60 min in CO_2_ incubator (pre-treatment incubation). After the pre-treatment, astrocytes were then incubated in ACSF, 100 mM K^+^ with Ca^2+^ free (FCHK-ACSF) containing the same agents of pre-treatment (20 min) and collection of the ACSF or FCHK-ACSF for analysis. Each 100 μL of collected ACSF or FCHK-ACSF was filtered by Vivaspin 500-3K (Sartorius, Goerringen, Germany) and freeze-dried for storage at −80 °C until needed for analyses. The composition of NaCl and KCl in fDMEM and ACSF were modified to maintain isotonicity and ionic strength [27,48,49,55,56,62].

After the sampling of astroglial transmitter releases, plasma membrane proteins of cultured astrocytes were extracted using Minute Plasma Membrane Protein Isolation Kit (Invent Biotechnologies, Plymouth, MN, USA). Plasma membrane fractions were solubilised by radio immunoprecipitation assay buffer (Fujifilm-Wako, Osaka, Japan) containing protease inhibitor cocktail (Nacalai Tesque, Kyoto, Japan) [50,54].

### 4.2. Ultra-High-Performance Liquid Chromatography (UHPLC)

L-glutamate levels were determined by using UHPLC equipped with xLC3185PU (Jasco, Tokyo, Japan) and fluorescence detection (xLC3120FP, Jasco, Tokyo, Japan) following dual derivatisation with isobutyryl-L-cysteine/o-phthalaldehyde. The derivatized samples (5 μL aliquots) were injected via an autosampler (xLC3059AS, Jasco, Tokyo, Japan). The analytical column (YMC Triat C18, particle 1.8 μm, 50 × 2.1 mm, YMC, Kyoto, Japan) was maintained at 45 °C, and the flow rate was set to 500 μL/min. A linear gradient elution program was used over a period of 10 min with mobile phases A (0.05 M citrate buffer, pH 5.0) and B (0.05 M citrate buffer containing 30% acetonitrile and 30% methanol, pH 3.5). The excitation/emission wavelengths of the fluorescence detector were set to 280/455 nm [29,59,60,86,87].

5-HT level were determined by UHPLC (xLC3185PU; Jasco) with electrochemical detection (ECD—300; Eicom, Kyoto, Japan) by a graphite carbon electrode set to +450 mV (vs. a Ag/AgCl reference electrode). The analytical column (Triart C18, particle 1.8 μm, 30 × 2.1 mm; YMC) was maintained at 40 °C, and the flow rate of the mobile phase was set at 500 μL/min. The mobile phase was made up of 0.1 M citrate buffer containing 1% methanol and 50 mg L^−1^ EDTA–_2_Na (final pH 6.0) [88,89].

### 4.3. Capillary Immunoblotting Analysis

The capillary immunoblotting analysis was performed, using Wes (ProteinSimple, Santa Clara, CA, USA), according to the ProteinSimple user manual. The lysates of the primary cultured astrocytes were mixed with a master mix (ProteinSimple) to a final concentration of 1 × sample buffer, 1 × fluorescent molecular weight marker, and 40 mM dithiothreitol and then heated at 95 °C for 5 min. The samples, blocking reagents (Immuno shot platinum, CosmoBio, Tokyo, Japan), primary antibodies, HRP-conjugated secondary antibodies, chemiluminescent substrate (SuperSignal West Femto: Thermo Fisher Scientific, Waltham, MA, USA), and separation and stacking matrices were also dispensed to the designated wells in a 25 well plate. After plate loading, the separation electrophoresis and immunodetection steps took place in the capillary system and were fully automated. A capillary immunoblotting analysis was carried out at room temperature, and the instrument’s default settings were used. Capillaries were first filled with a separation matrix followed by a stacking matrix, with about 40 nL of the sample used for loading. During electrophoresis, the proteins were separated by molecular weight through the stacking and separation matrices at 250 volts for 40–50 min and then immobilized on the capillary wall, using proprietary photo-activated capture chemistry. The matrices were then washed out. The capillaries were next incubated with a blocking reagent for 15 min, and the target proteins were immunoprobed with primary antibodies followed by HRP-conjugated secondary antibodies (Anti-Rabbit IgG HRP, A00098, 10 μg/mL, GenScript, Piscataway, NJ). The antibodies of GAPDH (NB300-322, 1:100, Novus Biologicals, Littleton, CO, USA), Cx43 (C6219, 1:100, Sigma-Aldrich, St. Louis, MO, USA), Erk (AF1576, 10 μg/mL, R&D systems, Minneapolis, MN, USA), pErk (AF1018, 5 μg/mL, R&D systems), 5-HT7R (NB100-56352, 1:50, Novus Biologicals) and 5-HT1AR (NBP2-21590, 1:100, Novus Biologicals) were diluted in an antibody diluent (Immuno shot plutinum) [83].

### 4.4. Data Analysis

All experiments in this study were designed with equally sized animal groups (*n* = 6), without carrying out a formal power analysis, in keeping with previous studies. All values are expressed as the mean ± SD, and *p* < 0.05 (two-tailed) was considered statistically significant for all tests. Drug levels in acute and subchronic administrations were selected based on values in previous studies [50,51,83,87]. Where possible, we sought to randomize and blind the data. In particular, for the determination of transmitter levels and protein expression, the sample order on the autosampler and Wes were determined by a random number table.

Concentration-dependent effects of acute and subchronic administrations of target agents on basal and FCHK-evoked astroglial L-glutamate releases were analysed by multivariate analysis of variance (MANOVA) using BellCurve for Excel ver. 3.2 (Social Survey Research Information Co., Ltd., Tokyo, Japan). When the data did not violate the assumption of sphericity (*p* > 0.05), the F-value of the MANOVA was analysed, using sphericity-assumed degrees of freedom. However, if the assumption of sphericity was violated (*p* < 0.05), the F-value was analysed, using Chi-Muller’s corrected degrees of freedom. When the F-value for the drug/time factors of MANOVA was significant, the data were analysed by a Tukey’s post hoc test.

Effects of acute and subchronic administrations of 5-HT receptor agents on basal and FCHK-evoked astroglial L-glutamate release were analysed by two-way analysis of variance (ANOVA) using BellCurve for Excel. When the F-value for the drug/time factors of ANOVA was significant, the data were analysed by a Tukey’s post hoc test. Interaction between vortioxetine and WAY100635 on the basal and FCHK-evoked astroglial L-glutamate release was analysed by one-way ANOVA using BellCurve for Excel. When the F-value for the drug/time factors of ANOVA was significant, the data were analysed by a Tukey’s post hoc test.

The effects of target agents on expression of Cx43, 5-HT1AR, 5-HT7R and phosphorylated Erk were analysed by student *t*-test using BellCurve for Excel. Interaction between vortioxetine and WAY100635 on expression of Cx43, 5-HT1AR, 5-HT7R and phosphorylated Erk was analysed by one-way ANOVA using BellCurve for Excel. When the F-value for the drug/time factors of ANOVA was significant, the data were analysed by a Tukey’s post hoc test.

### 4.5. Chemical Agents

Vortioxetine, escitalopram, the 5-HT1AR antagonist, WAY100635 and the 5-HT7R inverse agonist, SB269970, were obtained from Cosmo-Bio (Tokyo, Japan). The 5-HT1AR agonist BP554 was obtained from Fujifilm-Wako (Osaka, Japan). Escitalopram, WAY100635 and SB269970 were dissolved in fDMEM, ACSF or FCHK-ACSF directly. Vortioxetine and BP5449 were initially dissolved in dimethyl sulfoxide at 25 mM. The final dimethyl sulfoxide concentration was lower than 0.1% (*v*/*v*).

### 4.6. Nomenclature of Targets and Ligands

Key protein targets and ligands in this article are hyperlinked to corresponding entries in http://www.guidetopharmacology.org (15 September 2021), the common portal for data from the IUPHAR/BPS Guide to PHARMACOLOGY [90], and are permanently archived in the Concise Guide to PHARMACOLOGY [91].

## 5. Conclusions

The present study determined the concentration- and time-dependent effects of vortioxetine and escitalopram on astroglial L-glutamate release and the astroglial expression of 5-HT1AR, 5-HT7R, Cx43 and phosphorylated ERK, to explore the mechanisms of the antidepressive actions associated with tripartite synaptic transmission of escitalopram and vortioxetine. Neither acute nor subchronic administrations of escitalopram and WAY100635 under a 5-HT-free environment affected basal astroglial L-glutamate release and astroglial expressions of 5-HT1AR, 5-HT7R and Cx43 in the astroglial plasma membrane. In contrast to escitalopram, the subchronic administration of vortioxetine decreased the expressions of Cx43, 5-HT1AR, 5-HT7R and phosphorylated ERK in the plasma membrane, but increased Cx43 expression in the cytosol. Neither acute administrations of BP554, SB269970 nor vortioxetine affected astroglial L-glutamate release and astroglial expressions of 5-HT1AR, 5-HT7R and Cx43 in the plasma membrane; whereas the present study detected the effects of the subchronic administration of BP554, SB26970 and vortioxetine. The subchronic administration of BP554 (5-HT1AR agonist) and SB269970 (5-HT7R inverse agonist) downregulated the expression of 5-HT1AR, 5-HT7R and phosphorylated ERK in the plasma membrane. The subchronic administration of BP554 increased Cx43 expression in the cytosol without affecting Cx43 expression in the plasma membrane, whereas the subchronic administration of SB269970 decreased Cx43 expression in the plasma membrane without affecting Cx43 expression in the cytosol. Therefore, the downregulation of 5-HT1AR enhances Cx43 synthesis, but the downregulation of 5-HT7R suppresses Cx43 trafficking to the plasma membrane. The subchronic administration of vortioxetine inhibited astroglial basal L-glutamate release and astroglial L-glutamate release through activated hemichannels due to the suppression of functional Cx43 expression in the plasma membrane. These results that subchronic vortioxetine administration suppresses astroglial L-glutamate release induced by the trafficking of Cx43 to the astroglial plasma membrane suggest that astroglial transmission is probably a candidate for pathophysiological targets of depression.

## Figures and Tables

**Table 1:** Summary of the effects of acute and subchronic administrations of escitalopram (ESC), vortioxetine (VTX), BP554 (BP), WAY100635 (WAY) and SB269970 (SB) on astroglial L-glutamate release and protein expression of Cx43, 5-HT1AR, 5-HT7R and phosphorylated ERK in the astrocyte.

		ESC	VTX	VTX+WAY (vs. VTX)	WAY	BP	SB	Figure
(L-glutamate release)	Administration							
Basal	Acute	→	→	→(→)	→	→	→	Figure 1 and Figure 2
	Subchronic	→	↓	↓(→)	→	→	↓	Figure 1 and Figure 2
FCHK-Evoked	Acute	→	→	→(→)	→	→	→	Figure 1 and Figure 2
	Subchronic	→	↓	↓(→)	→	↓	↓	Figure 1 and Figure 3
**Protein Expression**	**Fraction**							
Cx43	Cytosol	→	↑	→ (↓)	→	↑	→	Figure 4
	Plasma membrane	→	↓	↓ (→)	→	→	↓	Figure 4
5-HT1AR	Plasma membrane	→	↓	↓ (↑)	→	↓	↓	Figure 5
5-HT7R	Plasma membrane	→	↓	↓ (→)	→	↓	↓	Figure 5
Phosphorylated ERK	Plasma membrane	→	↓	↓ (↑)	→	↓	↓	Figure 6

→: no effect, ↑: increased, ↓: decreased.

## Data Availability

The data presented in this study are available on request from the corresponding author. The data are not publicly available due to equipment dependent data.
